# Nitrogen-Rich Energetic Metal-Organic Framework: Synthesis, Structure, Properties, and Thermal Behaviors of Pb(II) Complex Based on *N*,*N*-Bis(1*H*-tetrazole-5-yl)-Amine

**DOI:** 10.3390/ma9080681

**Published:** 2016-08-10

**Authors:** Qiangqiang Liu, Bo Jin, Qingchun Zhang, Yu Shang, Zhicheng Guo, Bisheng Tan, Rufang Peng

**Affiliations:** 1 Research Center of Laser Fusion, China Academy of Engineering Physics, Mianyang 621010, China; liuqq006@mail.ustc.edu.cn; 2 State Key Laboratory Cultivation Base for Nonmetal Composites and Functional Materials, Southwest University of Science and Technology, Mianyang 621010, China; zhangqingchun@live.cn (Q.Z.); 410621b03kq.cdb@sina.cn (Y.S.); 3 School of National Defence Science and Technology, Southwest University of Science and Technology, Mianyang 621010, China; zcguo@swust.edu.cn; 4 Institute of Chemical Materials, Chinese Academy of Engineering Physics, Mianyang 621010, China; cqtbs@163.com

**Keywords:** nitrogen-rich materials, energetic materials, energetic MOFs

## Abstract

The focus of energetic materials is on searching for a high-energy, high-density, insensitive material. Previous investigations have shown that 3D energetic metal–organic frameworks (E-MOFs) have great potential and advantages in this field. A nitrogen-rich E-MOF, Pb(bta)·2H_2_O [N% = 31.98%, H_2_bta = *N*,*N*-Bis(1*H*-tetrazole-5-yl)-amine], was prepared through a one-step hydrothermal reaction in this study. Its crystal structure was determined through single-crystal X-ray diffraction, Fourier transform infrared spectroscopy, and elemental analysis. The complex has high heat denotation (16.142 kJ·cm^−3^), high density (3.250 g·cm^−3^), and good thermostability (*T*_dec_ = 614.9 K, 5 K·min^−1^). The detonation pressure and velocity obtained through theoretical calculations were 43.47 GPa and 8.963 km·s^−1^, respectively. The sensitivity test showed that the complex is an impact-insensitive material (IS > 40 J). The thermal decomposition process and kinetic parameters of the complex were also investigated through thermogravimetry and differential scanning calorimetry. Non-isothermal kinetic parameters were calculated through the methods of Kissinger and Ozawa-Doyle. Results highlighted the nitrogen-rich MOF as a potential energetic material.

## 1. Introduction

**Figure 1:**
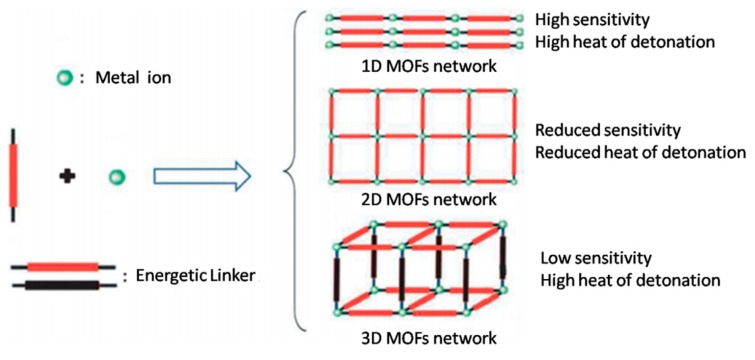
Design strategy for 1D, 2D, and 3D energetic MOFs (Reprinted from Refs. [28,49]. (Copyright 2013 and 2014 Wiley).

In the past decade, metal-organic frameworks (MOFs) have elicited much interest in chemistry, material science, medicine, and environmental science [1,2,3,4,5,6,7,8,9] because of their stable architectures, controllable structures, modifiable properties, and potential applications in gas storage [10,11,12,13], chemical separation [14,15,16,17], catalysis [18,19,20,21], chemical sensor technology [5], drug delivery [22,23,24], and so on. Many investigators have recently demonstrated the possibility of using nitrogen-rich MOFs as high explosives [25,26,27,28,29,30,31,32,33,34,35,36,37,38,39,40,41,42,43,44,45,46,47,48]. MOFs consist of metal ions (Pb^2+^, Ag^+^, etc.), energetic anions (e.g., N_3_^−^ and NO_3_^−^), or simple energetic ligands (triazole, tetrazole, tetrazine, hydrazine, etc.). The network structures of energetic MOFs (E-MOFs) can be designed as 1D, 2D, or 3D architectures (Figure 1) depending on the metal ion geometry and binding mode of the bridging energetic ligands.

**Figure 2:**
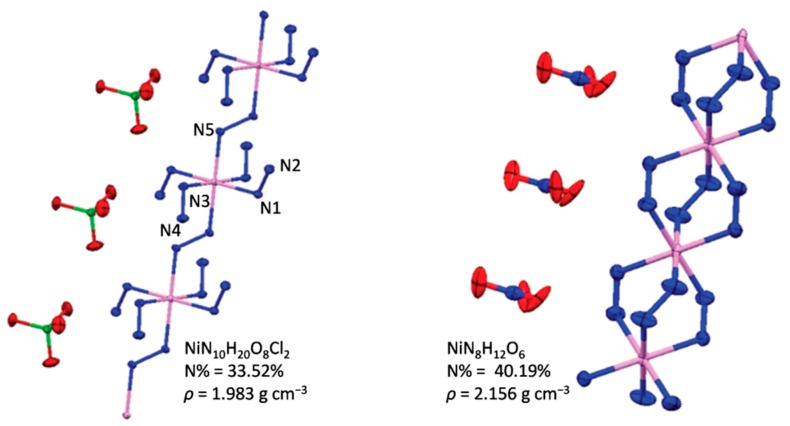
1D energetic MOFs of nickel hydrazine-perchlorate (NHP, **left**) and nickel hydrazine-nitrate (NHN, **right**) (Reprinted from Ref. [25]. Copyright 2012 American Chemical Society).

Several 1D E-MOFs (CHP: CoN_10_H_20_Cl_2_O_8_, N% = 33.49%, *ρ* = 1.948 g·cm^−3^, NHP: NiN_10_H_20_O_8_Cl_2_, NHN: NiN_8_H_12_O_6_; Figure 2) and 2D E-MOFs (CHHP: Co_4_C_4_H_48_N_24_O_26_Cl_4_, N% = 27.41%, *ρ* = 2.0 g·cm^−3^; ZnHHP: Zn_4_C_4_H_40_N_20_O_26_Cl_4_, N% = 23.57%, *ρ* = 2.117 g·cm^−3^) were developed by Hope-Weeks et al. through a combination of metal ion, energetic anion (ClO_4_^−^ and NO_3_^−^), and non-bridged ligand (hydrazine) [25,27]. 1D E-MOFs have high detonation heat but are very sensitive. The sensitivity and detonation heat of 2D E-MOFs are lower than those of 1D E-MOFs. Different from 1D linear and 2D layered structures, 3D frameworks possess more complicated connection modes, which could further enhance structural reinforcement and thus improve the stability and energetic properties [29,49]. In 2013, two 3D E-MOFs, namely, CuC_12_H_12_N_26_O_6_ (N% = 53.35%, *ρ* = 1.68 g·cm^−3^, IS = 22.5 J) and AgC_6_H_6_N_13_O_3_ (N% = 53.35%, *ρ* = 2.16 g·cm^−3^, IS = 30 J), were introduced by Pang et al. for the first time [28]. The sensitivities of these 3D MOFs are significantly lower than those of reported energetic coordination polymers, such as 1D (CHP, IS = 0.5 J) and 2D (ZnHHP, IS = 2.5 J; CHHP, IS = 0.8 J) MOFs. An increasing number of investigations on E-MOFs as new-generation high explosives were reported by Chen et al. [32,33,36,38,39,40,42,44,45,50,51], Pang et al. [28,48,52], Shreeve et al. [49,53], and so on [41,47,54,55,56] because of the advantages of 3D MOFs.

*N*,*N*-Bis(1*H*-tetrazole-5-yl)-amine (H_2_bta), a compound with high nitrogen content (N% = 82.34%), had its first single crystal structure and copper complexes reported by Klapötke et al. [57,58]. Shreeve et al. conducted extensive research on the energetic salts of H_2_bta [59,60,61] and found that the salts exhibit excellent energetic properties. The investigations also showed that H_2_bta may be an excellent energetic ligand to construct 3D MOFs for the following reasons [51]. First, the rigid structure of H_2_bta cannot only promote structural stability but can also improve the energetic performance of MOFs. Second, the versatile chelating-bridging coordination modes are propitious to the construction of high-dimensional 3D MOFs. Third, nitrogen atoms are involved in hydrogen-bond motifs to capture energetic moieties. Fourth, the predominant decomposition products are environmentally benign nitrogen gases. Fifth, the azide group hidden in aminotetrazole is the best stable moiety by virtue of the extended 6*π* system. Therefore, many MOFs based on H_2_bta, such as Cu(II) [39,62,63,64], Co(II,III) [36,50], Fe(III) [65], Mn(II) [66,67], Zn(II) [46,66,68,69], Cd(II) [66] MOFs, and others [70], have been obtained and investigated.

Pb(II)-bta MOFs (The Cambridge Crystallographic Data Centre (CCDC) numbers 650737, 721842, 721843) were synthesized originally via a two-step approach, and there were no reports about their energetic properties [71]. In this study, [Pb(bta)·2H_2_O]_n_ (CCDC number 1478651) was synthesized via a one-step hydrothermal reaction without any other assistant ligands (such as 2,2-bipyridine, 1,10-phenanthroline). The single crystal X-ray experiment revealedthe coordination mode of bta^2−^ with Pb^2+^. The energetic properties (detonation velocity, detonation pressure, and impact sensitivity) and thermal behavior of [Pb(bta)·2H_2_O]_n_ were also investigated. The thermodynamic parameters were obtained based on the reaction thermodynamic and kinetic equations. As expected, the complex exhibited high thermostability, excellent detonation properties, and acceptable sensitivity to impact. These featuressuggest potential applications as an energetic material.

## 2. Results and Discussion

General caution: H_2_bta and its derivatives are potentially explosive and should be handled in small quantities. Appropriate safety precautions should be taken, and larger scale synthesis is not recommended.

### 2.1. Synthesis of the Complex

**Scheme 1:**
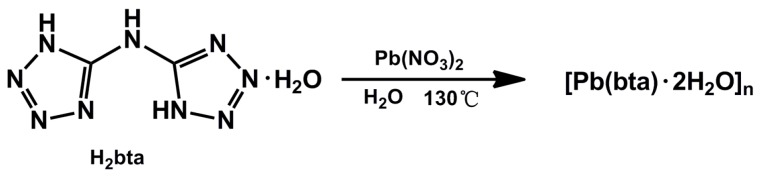
Synthesis of energetic 3D MOFs [Pb(bta)·2H_2_O]_n_.

H_2_bta·H_2_O was synthesized according to the literature [58]. Herein, three methods were used to synthesize Pb(II) coordination compounds based on H_2_bta, but only one route obtained the target complex successfully. As shown in Scheme 1, a mixture of Pb(NO_3_)_2_ (0.1 mmol) and H_2_bta·H_2_O (0.13 mmol) in H_2_O (4 mL) was sealed in a 10-mL Teflon-lined stainless autoclave and heated at 130 °C under autogenous pressure for three days and then cooled to room temperature over a further threedays. Colorless prismatic single-crystals suitable for X-ray diffraction were obtained.

### 2.2. Crystal Structure of the Complex

**Figure 3:**
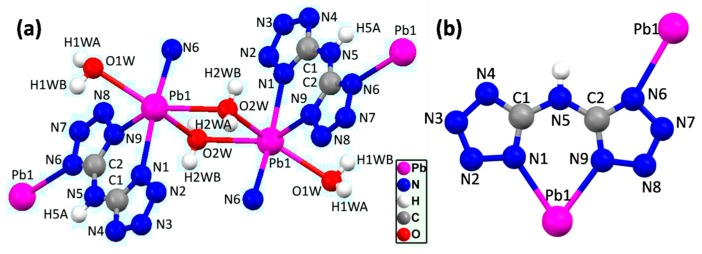
Ball-and-stick molecular structure of [Pb(bta)·2H_2_O] (**a**) and coordination mode of ligand (**b**).

**Figure 4:**
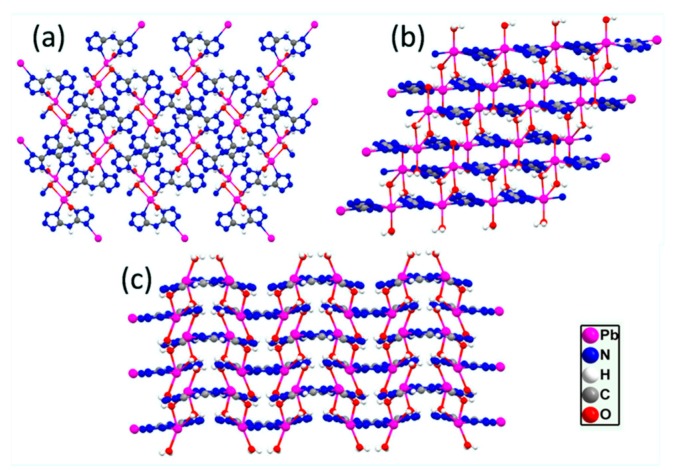
Ball-and-stick packing diagram of [Pb(bta)·2H_2_O]_n_ viewed down the *a*-axis(**a**); *b*-axis (**b**); and *a–c* diagonal (**c**).

**Figure 5:**
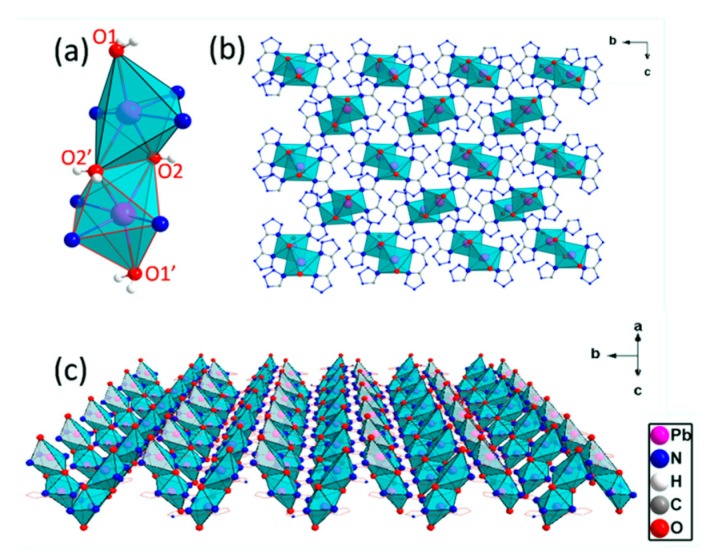
Coordination polyhedron geometry of [Pb(bta)·2H_2_O]_n_ (**a**) polyhedrons with Pb(II) as the center viewed down *a*-axis (**b**) and the *a–c* diagonal (**c**).

The X-ray crystallographic and structural refinement data are summarized in Table 1, and the structures are shown in Figure 3, Figure 4 and Figure 5. Further information on thecrystalstructure determination is providedin the Supplementary Materials (Tables S1–S6). Analysis of the X-ray crystallographic data for the complex shows that it crystallizes in the monoclinic space group *P*2_1_/*n* with a calculated density of 3.250 g·cm^−3^ based on four molecules packed in the unit-cell volume of 806.0(10) Å^3^. Density is a highly important physical property of energetic materials. Herein, because metal coordination can improve the densities of energetic materials, the density values of complex is much higher than that of the free ligand (1.693 g·cm^−3^).

The asymmetric unit is crystallographically independent with one Pb(II) ion, one bta^2−^ ligand, and two coordination water molecules (Figure 3a). Figure 3a shows that each Pb(II) ion is coordinated by three nitrogen atoms from two bta^2−^ ligands and three oxygen atoms from water molecules. Anirregular octahedral geometry is exhibited. Compared with the structure of H_2_bta [58], all of the bond lengths and angles are slightly changed, which may be caused by the negative charge on the anion rings and thecoordination environment. The C–N bond lengths of C1–N1 [1.322(9) Å], C1–N4 [1.322(9) Å], C1–N5 [1.379(9) Å], C2–N5 [1.377(8) Å], C2–N6 [1.330(8) Å], and C2–N9 [1.328(8) Å] are between the standard C–N single bond (1.47 Å) and standard C=N double bond (1.32 Å) lengths and are indicative of an aromatic system [72,73]. Meanwhile, the N–N bond lengths of N1–N2 [1.376(7) Å], N2–N3 [1.284(9) Å], N3–N4 [1.350(8) Å], N6–N7 [1.347(9) Å], N7–N8 [1.306(8) Å], and N8–N9 [1.369(8) Å] also fit between the standard N–N single bond (1.45 Å) and standard N=N double bond (1.25 Å) lengths [74,75], which further confirmstheconjugated and aromatic system. In addition, the bond angles C2–N5–C1 [125.0(6)°], N4–C1–N5 [112.8(6)°], N5–C2–N9 [127.2(6)°], and N6–C2–N9 [111.9(6)°] are slightly larger than thoseof H_2_bta because of the influence of the coordination environment. Figure 3b shows that the coordination mode of the ligand has three coordinated nitrogen atoms (N1, N9, and N6) in each bta^2−^. Atoms N1 and N9 in the ligand adopt chelating modes to connect to Pb(II) ion, whereas atom N6 adopts monodentate bridging modes to link with other Pb(II) ions.

The packing diagram of the complex viewed down the *a*-axis, *b*-axis, and the *a–c* diagonal isshown in Figure 4. The adjacent Pb(II) ions are bridged by two oxygen atoms from water in an antiparallel manner, with a Pb...Pb separation distance of 4.484 Å, Pb1–O1W distance of 2.670 Å, Pb1–O2W distance of 2.586 Å, O2W–Pb1–O2W angle of 69.01°, and O2W–Pb1–O1W angle of 144.05°. The view down the *b*-axis shows that a series of parallel rhombiare formed by the adjacent Pb1 and O2w. The torsion angles [i.e., N3–N2–N1–C1 (−0.2°), N9–Pb1–N1–N2 (179.2°), N1–N2–N3–N4 (−0.7°), and C2–N5–C1–N4 (179.3°)] are close to ±180° and 0°, which illustrates that Pb(II) and its chelating mode ligand are strictly coplanar. In addition, five types of hydrogen bonds [N5-H5A...N8^3#^ = 3.032 Å, O1W-H1WA...N2^4#^ = 2.841 Å, O1W-H1WB...N3^5#^ = 3.411 Å, O2W-H2WA...N3^6#^ = 2.856 Å, and O2W-H2WB...O1W^7#^ = 2.715 Å; symmetry transformations used to generate equivalent atoms: (^3#^ x − 1/2, −y + 3/2, z − 1/2),(^4#^ −x + 1/2, y − 1/2, −z + 3/2), (^5#^ −x, −y + 2, −z + 1), (^6#^ −x − 1/2, −y − 1/2, −z + 3/2), and (^7#^ x − 1, y, z)] (others are listed in the Supplementary Materials) exist in the target complex and further enhance the structural reinforcement. The complex [Pb(bta)·2H_2_O]_n_ has a good symmetrical and ordered 3D energetic framework.

The coordination polyhedron geometry of the complex is shown in Figure 5. Pb(bta)·2H_2_O is a six-coordinate complex, and its polyhedron with Pb(II) as the center is an irregular octahedral geometry. The basal plane of the octahedron is formed by three nitrogen atoms and O2’ atoms, and the vertexes are occupied by O1 and O2 atoms (Figure 5a). Two adjacent octahedrons share the O2...O2’ edge. Figure 5c shows that the planes that cross the Pb(II) ions in two interval polyhedrons are parallel. The two planes through the Pb(II) ions in two adjacent polyhedrons intersect, and the dihedral angle of the two planes is 33.8°.

### 2.3. Thermal Decomposition and Non-Isothermal Kinetics Analysis

#### 2.3.1. Thermal Decomposition

**Figure 6:**
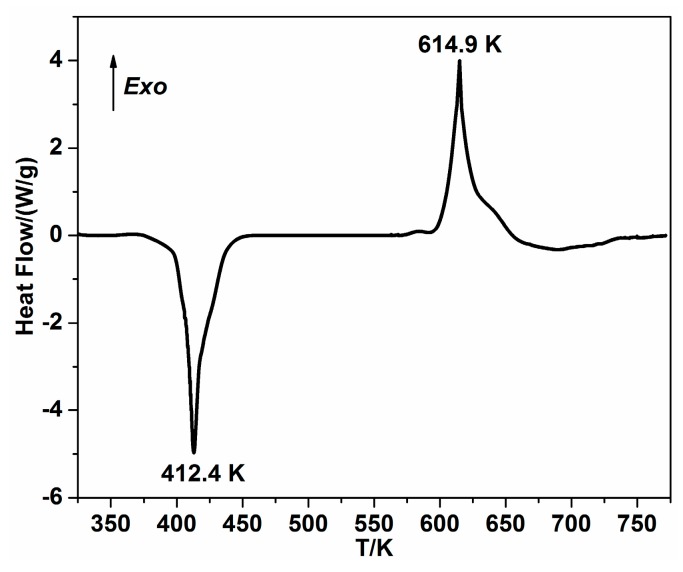
DSC curve of [Pb(bta)·2H_2_O]_n_ at a heating rate of 5 K·min^−1^.

Approximately 1.0 mg of the complex was tested through differential scanning calorimetry (DSC) in an open crucible at a heating rate of 5 K·min^−1^ under nitrogen atmosphere to determine the melting points and decomposition temperatures. One endothermic process (loss of crystal water) peak and one exothermic process (decomposition) peak at 412.4 K and 614.9 K, respectively, are visible in the DSC curve (Figure 6). The relevant exothermic enthalpy change of the compound is 352.1 kJ·mol^−1^. Therefore, this metal–organic crystal containing the bta^2−^ ligand possesses sufficient thermal stability to be an energetic material. In addition, the effect on *T*_p_ by the particle size were also investigated (Figures S2 and S3). The thermal behavior of millimeter sized single crystals and micron sized crystals were studied by DSC at the heating rate of 10 K·min^−1^ under nitrogen atmosphere. The peak decomposition temperature of micron sized crystals MOF (about 10 μm) is 615.4 K, which is lower than that of single crystals MOF (*T*_p_ = 620.1 K). The results are consistent with the reference by Cacho-Bailo et al. [76].

**Figure 7:**
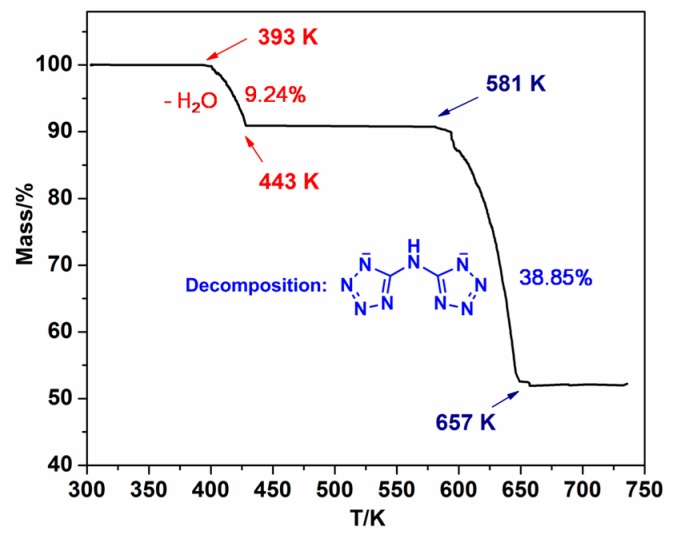
TGA curve of [Pb(bta)·2H_2_O]_n_ at a heating rate of 5 K·min^−1^.

The decomposition process of the complex was also investigated through the rmogravimetric analysis (TGA) at a heating rate of 5 K·min^−1^ in nitrogen atmosphere. Figure 7 shows that the decomposition process of the complex can be divided into two steps, and the total mass loss is 48.09%. The first process in the range of 393 K–443 K was confirmed as the loss of crystal water (observed 9.24%, calculated 9.13%). This result indicates that the water molecules in the title complex were stable before 443 K. The second process from 581 to 657 K was considered the collapse of the structures related to the nitrogen-rich bta^2−^ groups.

#### 2.3.2. Non-Isothermal Kinetics Analysis

**Figure 8:**
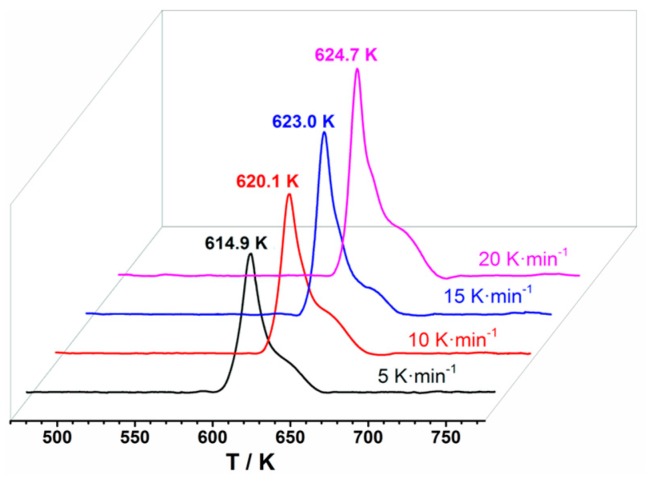
DSC curves of [Pb(bta)·2H_2_O]_n_ at different heating rate.

Kissinger’s [77] and Ozawa’s methods [78,79] were used to determine the kinetics parameters based on the exothermic peaks temperature measured from DSC curves with four different heating rates (5, 10, 15, 20 K·min^−1^; Figure 8).

Using the values of peak temperature, Kissinger Equation (Equation (1) in Section 3) and Ozawa-Doyle (Equation (2) in Section 3), the apparent activation energy (*E*) and pre-exponential factor (*A*) were calculated. The calculated results including linear correlation coefficient *r* are shown in Table 2. The results show that the value of *E* (about 430 kJ·mol^−1^) is higher than that of 1,3,5-trinitrohexahydro-1,3,5-triazine (RDX, about 142 kJ·mol^−1^) [62], cyclotetramethylene tetranitramine (HMX, about 238 kJ·mol^−1^) [62] and 2,4,6,8,10,12-hexanitro-2,4,6,8,10,12-hexaazatetracyclo [5.5.0.0.0] dodecane (CL-20, about 200 kJ·mol^−1^) [80,81], which is in accordance with its excellent thermostability.

### 2.4. Energetic Properties

#### 2.4.1. Heat Detonation of Complex

We selected anidentical method for CHP and NHPto estimate the detonationheat (∆*H*_det_) of the compound and compare it withthevalues for E-MOFs and classical energetic materials [25]. For the complex, Pb, N_2_, H_2_O, NH_3_, and C were assumed to be the final decomposition products of the organic part of the framework, and all non-metal-containing products, including water, were regardedas gas. The detonation reaction considered for the compound is described by Equation (3) (see Section 3), and the heat detonation value was obtained with Equation (4) (see Section 3), which was developed from known ∆*H*_det_ data for 11 commonly used high explosives.

**Figure 9:**
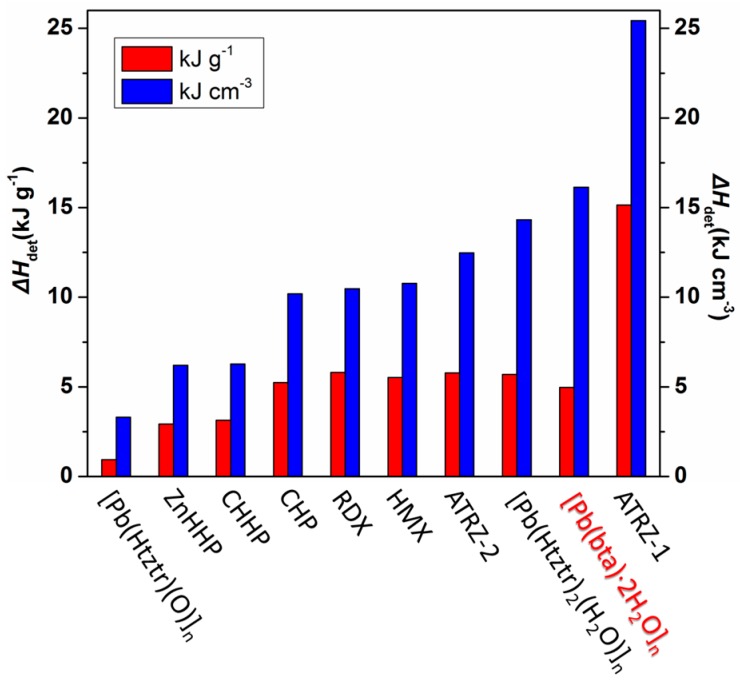
Bar chart representation of ∆*H*_det_ values in literature for common explosive materials and previously reported values for energetic MOFs along with the predicted ∆*H*_det_ value for [Pb(bta)·2H_2_O]_n_. The errorbars correspond to the 96% statistical-confidence level for these values.

Heat of detonation (∆*H*_det_) was calculated to be 4.966 kJ·g^−1^. It is higher than those of MOFs ([Pb(Htztr)(O)]_n_, 0.94 kJ·g^−1^; CHHP, 3.14 kJ·g^−1^; ZnHHP, 2.93 kJ·g^−1^) but lower than those of RDX (5.799 kJ·g^−1^) and HMX (5.523 kJ·g^−1^) (see in Table 3). Figure 9 shows that despite the lack of advantage of the ∆*H*_det_ of the per gram complex, the ∆*H*_det_ of per cm^3^ (16.142 kJ·cm^−3^) is higher than that of traditional explosives and E-MOFs, except for ATRZ-1 (ATRZ: 4,4’-azo-1,2,4-triazole) because of its high density.

#### 2.4.2. Detonation Properties and Sensitivity

The performance of a high explosiveischaracterized by its detonation velocity, *D* (km·s^−1^), and detonation pressure, *P* (GPa). The *D* and *P* of the complex were calculated with the Kamlet–Jacobs equations (see Equations (5)–(7) in Section 3), which are usually applied to E-MOFs reported previously. Table 3 shows a comparison of the physicochemical properties of several energetic materials and the complex. The *D* and *P* of the complex are 8.963 km·s^−1^ and 47.47 GPa, respectively. Its *D* is higher than that of HMX (8.900 km·s^−1^), RDX (8.600 km·s^−1^), and other E-MOFs (6.205–8.226 km·s^−1^), except for ATRZ-1 (9.160 km·s^−1^).

Sensitivity deserves significant attention from researchers because it is closely linked with the safety of handling and applying explosives. The impact sensitivity (IS) of the compound was investigated for initial safety testing. Table 3 provides a summary of the data collected. The IS of [Pb(bta)·2H_2_O]_n_ is more than 40 J, whereas the IS of RDX is 7.4 J under the same test condition. The IS of the complex is more insensitive than traditional explosives (HMX, 7.4 J; RDX, 7.4J) and reported energetic coordination polymers, such as 1D (CHP, IS = 0.5 J), 2D (ZnHHP, IS = 2.5 J; CHHP, IS = 0.8 J), and 3D (ATRZ-1, IS = 22.5 J) MOFs. These results reveal that the compound is insensitive to external stimuli because of the stabilized 3D covalent framework, in which the molecules are more rigid than those in 1D or 2D structures. Thus, [Pb(bta)·2H_2_O]_n_ can be classified as an impact-insensitive energetic material.

## 3. Materials and Methods

The FT-IR spectrum was recorded on Nicolet 380 FT-IR spectrophotometer (Thermo Fisher Nicolet, Waltham, MI, USA) employing a KBr matrix with a resolution of 4 cm^−1^, in the wavelength range of 400 cm^−1^ to 4000 cm^−1^. Elemental analysis was performed on a Vario ELCUBE Elemental Analyzer (Elementar, Hanau, Germany). DSC was performed by a Q200 DSC instrument (TA Instruments, New Castle, DE, USA) at a heating rate of 5, 10, 15 and 20 K·min^−1^, respectively, in flowing high-purity nitrogen. Approx. 1.0 mg sample was sealed in aluminum pans in the temperature range of 313 to 773 K for DSC experiments. The sensitivity to impact stimuli was determined by fall hammer apparatus according tothe standard staircase method (GJB of China). With a step of 0.04 cm, 50 ± 1 mg of test specimens were used and a 10-kg drop weight was allowed to fall freely from different heights. The results were reported in terms of height for 50% probability of explosion (*h*_50*%*_). The picture of micron sized crystal MOF was obtained by a microscope (×1000). Molecular structure packing diagram and coordination polyhedron geometry of [Pb(bta)·2H_2_O]_n_ were drawn by Mercury and Diamond software (Diamond 3.1, Crystal Impact GbR, Bonn, Germany).

### 3.1. Synthesis of [Pb(bta)·2H_2_O]_n_

Method 1: a mixture of Pb(NO_3_)_2_ (33 mg, 0.1 mmol) and H_2_bta·H_2_O (22 mg, 0.13 mmol) in H_2_O (4 mL) was sealed in a 10-mL Teflon-lined stainless autoclave and heated at 200 °C under autogenous pressure for 3 days and then cooled to room temperature over a further 3 days. Two colorless single-crystals (needle and prismatic crystals) were picked up and a single crystal test indicated they are two different crystals of Pb(N_3_)_2_. See the structures in Supplementary Materials (Figure S1, Tables S8–S12).

Method 2: a suspension of H_2_O (8 mL) and Pb(II) complex (30 mg), which was obtained from Na_2_bta and Pb(NO_3_)_2_ as raw materials by means of metathesis reaction, was sealed in a 10 mL Teflon-lined stainless autoclave. Both experiments were heated at 130 °C and 200 °C, respectively, under autogenous pressure for 3 days and then cooled to room temperature over a further 3 days. Unfortunately, there were powder instead of crystals in Teflon autoclave.

Method 3: a mixture of Pb(NO_3_)_2_ (33 mg, 0.1 mmol) and H_2_bta·H_2_O (22 mg, 0.13 mmol) in H_2_O (4 mL) was sealed in a 10-mL Teflon-lined stainless autoclave and heated at 130 °C under autogenous pressure for 3 days and then cooled to room temperature over a further 3 days. Colorless prismatic single-crystals were picked up and yield 23.8 mg (61% based on Pb). DSC (5 K·min^−1^): 614.9 K(dec.). FT-IR (KBr) *ṽ*: 3432(vs), 3292(s), 2921(m), 2854(m), 1623(vs), 1525(m), 1500(m), 1420(m), 1314(m), 1230(w), 1127(w), 1115(w), 1069(w), 1015(w), 794(m), 740(m). Elemental analysis (C_2_H_5_N_9_O_2_Pb, 394.34) Calcd: C 6.09%, H 1.28%, N 31.98%; Found: C 6.11%, H 1.30%, N 31.89%.

### 3.2. Single-Crystal X-ray Diffraction Analyses

The single-crystal X-ray experiments were performed on a Smart Apex CCD diffractometer (Bruker) (Bruker, Karlsruhe, Germany) equipped with graphite monochromatized Mo *Kα* radiation (*λ* = 0.71073 A) using the *ω* and *ϕ* scan mode. The structure was solved by direct methods using SHELXS-97 (Göttingen, Germany) [82] and refined by means of full-matrix least-squares procedures on *F*^2^ with the SHELXL-97 program [83]. All non-H atoms were located using subsequent Fourier-difference methods and refined anisotropically. In all cases, hydrogen atoms were placed in their calculated positions and thereafter allowed to ride on their parent atoms.

### 3.3. Equations for Calculating Non-Isothermal Kinetics

The Kissinger (1) and Ozawa-Doyle (2) Equations are as follows:

(1) $$\ln[\beta /{T_{p}}^{2}]=\ln[A\text{R}/E_{\text{a}}]-[E_{\text{a}}/\text{R}T_{p}],$$

(2) $$\mathrm{lg}\beta =\text{C}-0.4567E_{a}/\text{R}T,$$

where *β* is the heating rate; *T*_p_ is the peak temperature; *A* is the pre-exponential factor; *E* is the apparent activation energy; and R is the gas constant (8.314 J·K^−1^·mol^−1^). Linear relationship of ln(*β*/*T_p_*^2^) and lg (*β*) vs. 1/*T*_p_ are shown in Supplementary Materials (Figure S4).

### 3.4. Calculation for Heat of Detonation

The complete detonation reactions are described by Equation (3). According to Ref. [25], Density Functional Theory (DFT) was used to calculate the energy of detonation (Δ*E*_det_), from which Δ*H*_det_ was estimated by using a linear correlation Equation (4), and the calculated parameters was listed in Table S7. The DFT calculation for [Pb(bta)·2H_2_O]_n_ was performed with the code DMOl3 [84] under 3D periodic boundary conditions employing the Monkhorst–Pack multiple *K*-point sampling of the Brillouin zone [85] and the Perdew–Becke–Ezerhoff (PBE) exchange-correlation function [86]:

(3) $${\text{C}}_{2}{\text{H}}_{5}{\text{N}}_{9}{\text{O}}_{2}\text{Pb}\to \text{Pb}+\frac{13}{3}{\text{N}}_{2}+2{\text{H}}_{2}\text{O}+\frac{1}{3}\text{N}{\text{H}}_{3}+2\text{C},$$

(4) $$\Delta H_{\text{det}}=1.127\;\Delta E_{\text{det}}+\;0.046.$$

### 3.5. Calculation for Detonation Velocity and Detonation Pressure

The *D* and *P* of the complex were calculated by Kamlet–Jacbos Equations [28] as follows, which were usually applied to the E-MOFs reported previously:

(5) $$D=1.01\Phi^{1/2}(1+1.30\rho ),$$

(6) $$P=1.558\Phi \rho^{2},$$

(7) $$\Phi =31.68N{\left(MQ\right)}^{1/2}.$$

where *D* is detonation velocity (km·s^−1^); *P* is detonation pressure (GPa); *N* is moles of detonation gases per gram of explosive; *M* is average molecular weight of the gases; *Q* is heat of detonation (kcal·g^−1^); *ρ* is density of explosive (g·cm^−3^); and *Φ* is a parameter determined by *N*, *M* and *Q*.

## 4. Conclusions

We successfully synthesized a nitrogen-rich E-MOF, namely, Pb(bta)·2H_2_O [N% = 31.98%, H_2_bta = *N*,*N*-Bis(1*H*-tetrazole-5-yl)-amine]. It was characterized through various techniques, such as elemental analyses, Fourier transform infrared spectroscopy, TG, DSC, and single crystal X-ray diffraction. X-ray single crystal structure analysis showed that the crystal of the complex in the monoclinic space group *P*2_1_/*n* has a calculated density of 3.250 g·cm^−3^. The DSC curve indicated that it has good thermostability. One endothermic process (around 412.4 K) and one exothermic process (around 614.9 K) exist at the heating rate 5 K·min^−1^. The calculated results showed that the detonation heat, detonation pressure, and velocity are 4.966 kJ·g^−1^ (16.142 kJ·cm^−3^), 43.47 GPa, and 8.963 km·s^−1^, respectively. The sensitivity test showed that the complex is an impact-insensitive material (IS > 40 J). The thermal decomposition process and kinetic parameters of the complex were also investigated through TG and DSC. The non-isothermal kinetic parameters were calculated withthe methods of Kissinger and Ozawa-Doyle. The activation energy value (about 430 kJ·mol^−1^) is higher than that of RDX, HMX, and CL-20. Excellent impact sensitivity and high thermal stability depend on good detonation properties. The 3D MOF in this study has potential applications as an energetic material.

## Figures and Tables

**Table 1:** Crystal data and structure refinement data of [Pb(bta)·2H_2_O]_n_.

Empirical Formula	C_2_H_5_N_9_O_2_Pb
Formula weight	394.34
Crystal Color	colorless
Crystal size (mm^3^)	0.21 × 0.20 × 0.19 mm
Crystal system	Monoclinic
Space group	*P*2_1_/*n*
*a* (Å)	6.592(5)
*b* (Å)	11.987(9)
*c* (Å)	10.552(8)
*α* (°)	90
*β* (°)	104.856(12)
*γ* (°)	90
*V* (Å^3^)	806.0(10)
*Z*	4
*ρ*_calcd_ (g·cm^−3^)	3.250
*T* (K)	150(2)
*F* (000)	712
*θ* (°)	2.62 to 25.00
*R*_int._	0.0425
Data	1423
Restraints	4
parameters	127
GOF ^a^ on *F*^2^	1.051
*R*_1_^b^ (*I* > 2*σ* (*I*))	0.0241
*ωR*_2_^c^ (I > 2*σ* (*I*))	0.0655
*R*_1_ (all data)	0.0261

^a^ GOF = Goodness of Fit; ^b^ *R*_1_ = ∑||*F*_o_| − |*F*_c_||/∑|*F_o_*|; ^c^ *ωR*_2_ = [(*ω*(*F*_o_^2^ − *F*_c_^2^)^2^)/*ω*(*F*_o_^2^)^2^]^1/2^.

**Table 2:** The calculated kinetic parameters for the exothermic decomposition processes of [Pb(bta)·2H_2_O]_n_.

*β*/(K·min^−1^)	T_p_/(K)	Kissinger			Ozawa-Doyle	
		*E*/(kJ·mol^−1^)	ln *A*	*r*	*E*/(kJ·mol^−1^)	*r*
5	614.9	436.1	84.93	0.9984	424.6	0.9984
10	620.1					
15	623.0					
20	624.7					

**Table 3:** Physicochemical properties of [Pb(bta)·2H_2_O]_n_.

	*T*_d_^a^	*p* ^b^	N ^c^	Δ*H*_det_^d^	*D* ^e^	*P* ^f^	IS ^g^
[Pb(bta)·2H_2_O]_n_	342	3.250	31.98	4.97	8.963	47.47	>40.0
CHP ^h^ [25]	194	1.948	14.71	5.23	8.225	31.73	0.5
CHHP ^h^ [27]	231	2.000	28.25	3.14	6.205	17.96	0.8
ZnHHP ^h^ [27]	293	2.117	23.61	2.93	7.016	23.58	2.5
ATRZ-1 ^h^ [29]	243	1.680	53.35	15.14	9.160	35.68	22.5
ATRZ-2 ^h^ [29]	257	2.160	43.76	5.78	7.773	29.70	30.0
[Pb(Htztr)_2_^h^ (H_2_O)]_n_ [32]	340	2.519	39.40	5.69	7.715	31.57	>40.0
[Pb(Htztr)(O)]_n_^h^ [32]	318	3.511	27.20	0.94	8.122	40.12	>40.0
HMX [25]	287	1.950	37.84	5.52	8.900	38.39	7.4
RDX [25]	210	1.806	37.80	5.80	8.600	33.92	7.4

^a^ Decomposition temperature; ^b^ Density from X-ray diffraction analysis (g·cm^−3^); ^c^ Nitrogen content (%); ^d^ The heat of detonation (kJ·g^−1^); ^e^ Detonation velocity (km·s^−1^); ^f^ Detonation pressure (GPa); ^g^ Impact sensitivity; ^h^ CHP = cobalt hydrazine perchlorate; CHHP = cobalt hydrazine hydrazinecarboxylate perchlorate; ZnHHP = zinc hydrazine hydrazinecarboxylate perchlorate; ATRZ = 4,4’-azo-1,2,4-triazole; Htztr = 3-(tetrazol-5-yl)triazole.

## Supplementary Materials

The following are available online at www.mdpi.com/1996-1944/9/8/681/s1. Figure S1: Ball-and-stick molecular structure and packing diagram of Pb(N_3_)_2_, Figure S2: The pictures of single crystal MOF and micron sized crystal MOF, Figure S3: DSC curves of single crystal MOFand micron sized crystal MOFat the heating rate of 10 K·min^−1^, Figure S4: Linear relationship of ln *(β*/*T_p_*^2^) and lg (*β*) vs. 1/*T_p_*, Table S1: Atomic coordinates and equivalent isotropic displacement parameters for [Pb(bta)·2H_2_O]_n_, Table S2: Bond lengths and angles for [Pb(bta)·2H_2_O]_n_, Table S3: Anisotropic displacement parameters for [Pb(bta)·2H_2_O]_n_, Table S4: Hydrogen coordinates and isotropic displacement parameters for [Pb(bta)·2H_2_O]_n_, Table S5: Torsion angles for [Pb(bta)·2H_2_O]_n_, Table S6: Hydrogen bonds for [Pb(bta)·2H_2_O]_n_, Table S7: Calculated parameters used in the detonation reactions, Table S8: Crystal data and structure refinement for Pb(N_3_)_2_, Table S9: Atomic coordinates and equivalent isotropic displacement parameters for Pb(N_3_)_2_, Table S10: Bond lengthsand anglesfor Pb(N_3_)_2_, Table S11: Anisotropic displacement parameters for Pb(N_3_)_2_, Table S12: Torsion angles for Pb(N_3_)_2_.
