# Supplementary material for: Nitrogen-Rich Energetic Metal-Organic Framework: Synthesis, Structure, Properties, and Thermal Behaviors of Pb(II) Complex Based on N,N-Bis(1H-tetrazole-5-yl)-Amine
Source: Materials (Basel). 2016 Aug 10;9(8):681. doi: 10.3390/ma9080681 (PMC5512347; doi:10.3390/ma9080681)
Supplement: Supplementary file 1 [file materials-09-00681-s001.pdf]

Supplementary Materials: Nitrogen-Rich Energetic Metal-Organic Framework:Synthesis, Structure, Properties, and Thermal Behaviors of Pb(II) Complex Based on *N*,*N*-Bis(1*H*-tetrazole-5-yl)-amine

Qiangqiang Liu, Bo Jin, Qingchun Zhang, Yu Shang, Zhicheng Guo, Bisheng Tan and Rufang Peng

Table S1. Atomic coordinates (×10^4) and equivalent isotropic displacement parameters (Å^2×10^3) for [Pb(bta)·$2H_2O$]$_n$. U(eq) is defined as one third of the trace of the orthogonalized U_{ij} tensor.

	x	y	z	U(eq)
Pb(1)	1932(1)	9485(1)	8633(1)	13(1)
N(2)	1253(10)	11430(5)	6708(6)	21(1)
N(1)	862(10)	10301(4)	6783(6)	17(1)
N(3)	2507(9)	11663(5)	5593(6)	20(1)
N(5)	2096(9)	8827(5)	5234(6)	16(1)
C(1)	1984(10)	9923(6)	5641(7)	14(1)
N(4)	3001(10)	10729(5)	4864(6)	18(1)
C(2)	1113(10)	7945(5)	5981(6)	9(1)
O(1W)	4971(8)	8666(4)	7647(5)	21(1)
O(2W)	1314(8)	8958(4)	9457(5)	20(1)
N(9)	202(8)	7992(4)	7167(5)	12(1)
N(8)	785(10)	6908(5)	7461(6)	20(1)
N(6)	1385(10)	6906(5)	5530(6)	19(1)
N(7)	182(10)	6273(5)	6487(6)	24(1)

Table S2. Bond lengths (Å) and angles (°) for [Pb(bta)·$2H_2O$]$_n$.

Bonds/Angles	Length(Å)/Angle(°)
Pb(1)-N(9)	2.447(5)
Pb(1)-N(1)	2.513(6)
Pb(1)-O(2W)	2.586(5)
Pb(1)-N(6)$^{\#1}$	2.623(6)
Pb(1)-O(1W)	2.670(5)
N(2)-N(3)	1.284(9)
N(2)-N(1)	1.376(7)
N(1)-C(1)	1.322(9)
N(3)-N(4)	1.350(8)
N(5)-C(2)	1.377(8)
N(5)-C(1)	1.379(9)
N(5)-H(5A)	0.8800
C(1)-N(4)	1.332(9)
C(2)-N(9)	1.328(8)
C(2)-N(6)	1.330(8)
O(1W)-H(1WB)	0.8920
O(1W)-H(1WA)	0.8417
O(2W)-H(2WB)	0.8497
O(2W)-H(2WA)	0.9339
N(9)-N(8)	1.369(8)
N(8)-N(7)	1.306(8)
N(6)-N(7)	1.347(9)

Table S2. *Cont.*

Bonds/Angles	Lengths (Å)/Angles (°)
N(6)-N(7)	1.347(9)
N(6)-Pb(1)#2	2.623(6)
N(9)-Pb(1)-N(1)	70.33(18)
N(9)-Pb(1)-O(2W)	75.08(18)
N(1)-Pb(1)-O(2W)	81.5(2)
N(9)-Pb(1)-N(6)#1	93.15(19)
N(1)-Pb(1)-N(6)#1	157.3(2)
O(2W)-Pb(1)-N(6)#1	79.20(19)
N(9)-Pb(1)-O(1W)	76.09(17)
N(1)-Pb(1)-O(1W)	108.31(19)
O(2W)-Pb(1)-O(1W)	144.05(16)
N(6)#1-Pb(1)-O(1W)	81.42(18)
N(3)-N(2)-N(1)	109.5(6)
C(1)-N(1)-N(2)	103.4(6)
C(1)-N(1)-Pb(1)	134.6(4)
N(2)-N(1)-Pb(1)	121.1(4)
N(2)-N(3)-N(4)	110.2(5)
C(2)-N(5)-C(1)	125.0(6)
C(2)-N(5)-H(5A)	117.5
C(1)-N(5)-H(5A)	117.5
N(1)-C(1)-N(4)	112.8(6)
N(1)-C(1)-N(5)	125.7(6)
N(4)-C(1)-N(5)	121.5(6)
C(1)-N(4)-N(3)	104.0(6)
N(9)-C(2)-N(6)	111.9(6)
N(9)-C(2)-N(5)	127.2(6)
N(6)-C(2)-N(5)	120.9(6)
Pb(1)-O(1W)-H(1WB)	101.4
Pb(1)-O(1W)-H(1WA)	125.0
H(1WB)-O(1W)-H(1WA)	93.6
Pb(1)-O(2W)-H(2WB)	96.7
Pb(1)-O(2W)-H(2WA)	105.8
H(2WB)-O(2W)-H(2WA)	124.5
C(2)-N(9)-N(8)	104.5(5)
C(2)-N(9)-Pb(1)	135.4(4)
N(8)-N(9)-Pb(1)	119.5(4)
N(7)-N(8)-N(9)	108.8(5)
C(2)-N(6)-N(7)	105.0(5)
C(2)-N(6)-Pb(1)#2	149.4(4)
N(7)-N(6)-Pb(1)#2	105.5(4)
N(8)-N(7)-N(6)	109.7(5)

Symmetry transformations used to generate equivalent atoms: #1 x+1/2,−y+3/2,z+1/2; #2 x−1/2,−y+3/2,z−1/2.

Table S3. Anisotropic displacement parameters (Å^2× 10^3) for [Pb(bta)·2H_2O]$_n$. The anisotropic displacement factor exponent takes the form: −2 π^2 [h^2 a^{*2} U^{11} + ... + 2 h k a* b* U^{12}].

	U11	U22	U33	U23	U13	U12
Pb(1)	13(1)	11(1)	13(1)	0(1)	1(1)	−1(1)
N(2)	26(3)	11(3)	24(3)	1(3)	2(3)	8(3)
N(1)	26(3)	5(3)	18(3)	−1(2)	−1(3)	4(2)
N(3)	18(3)	10(3)	32(4)	4(3)	9(3)	3(2)
N(5)	18(3)	10(3)	14(3)	−2(2)	−7(2)	2(2)
C(1)	11(3)	13(3)	19(4)	2(3)	4(3)	1(3)
N(4)	21(3)	15(3)	15(3)	2(2)	−2(3)	1(2)
C(2)	8(3)	11(3)	8(3)	−2(2)	3(3)	−3(2)
O(1W)	24(3)	18(3)	17(3)	2(2)	1(2)	2(2)
O(2W)	21(3)	17(2)	21(3)	−1(2)	4(2)	−5(2)
N(9)	11(3)	9(3)	15(3)	−1(2)	1(2)	2(2)
N(8)	23(3)	14(3)	19(3)	−1(2)	0(3)	4(2)
N(6)	22(3)	15(3)	19(3)	−3(2)	1(3)	0(2)
N(7)	28(4)	14(3)	25(4)	−2(3)	1(3)	4(3)

Table S4. Hydrogen coordinates (×10^4) and isotropic displacement parameters (Å^2× 10^3) for [Pb(bta)·2H_2O]$_n$.

	x	y	z	U(eq)
H(5A)	−2854	8679	4436	19
H(1WB)	4430	8773	6790	31
H(1WA)	5198	7983	7557	31
H(2WB)	−2233	9129	8760	30
H(2WA)	−1066	8235	9790	30

Table S5. Torsion angles (°) for [Pb(bta)·2H_2O]$_n$.

Torsion Angles	Angle(°)
N(3)-N(2)-N(1)-C(1)	−0.2(8)
N(3)-N(2)-N(1)-Pb(1)	170.4(5)
N(9)-Pb(1)-N(1)-C(1)	−13.6(7)
O(2W)-Pb(1)-N(1)-C(1)	−90.8(7)
N(6)$^{\#1}$-Pb(1)-N(1)-C(1)	−59.0(10)
O(1W)-Pb(1)-N(1)-C(1)	53.6(7)
N(9)-Pb(1)-N(1)-N(2)	179.2(6)
O(2W)-Pb(1)-N(1)-N(2)	102.1(5)
N(6)$^{\#1}$-Pb(1)-N(1)-N(2)	133.9(5)
O(1W)-Pb(1)-N(1)-N(2)	−113.6(5)
N(1)-N(2)-N(3)-N(4)	−0.7(8)
N(2)-N(1)-C(1)-N(4)	1.1(8)
Pb(1)-N(1)-C(1)-N(4)	−167.6(5)
N(2)-N(1)-C(1)-N(5)	179.3(6)
Pb(1)-N(1)-C(1)-N(5)	10.6(11)
C(2)-N(5)-C(1)-N(1)	2.7(11)
C(2)-N(5)-C(1)-N(4)	−179.3(7)
N(1)-C(1)-N(4)-N(3)	−1.5(8)
N(5)-C(1)-N(4)-N(3)	−179.8(6)
N(2)-N(3)-N(4)-C(1)	1.3(8)
C(1)-N(5)-C(2)-N(9)	−4.7(11)
C(1)-N(5)-C(2)-N(6)	176.9(7)

Table S5. *Cont.*

Torsion Angles	Angles (°)
N(6)-C(2)-N(9)-N(8)	0.3(7)
N(5)-C(2)-N(9)-N(8)	−178.2(6)
N(6)-C(2)-N(9)-Pb(1)	171.5(5)
N(5)-C(2)-N(9)-Pb(1)	−7.0(11)
N(1)-Pb(1)-N(9)-C(2)	11.8(6)
O(2W)-Pb(1)-N(9)-C(2)	97.9(6)
N(6)[#1]-Pb(1)-N(9)-C(2)	175.9(6)
O(1W)-Pb(1)-N(9)-C(2)	−103.8(6)
N(1)-Pb(1)-N(9)-N(8)	−177.9(5)
O(2W)-Pb(1)-N(9)-N(8)	−91.8(5)
N(6)[#1]-Pb(1)-N(9)-N(8)	−13.9(5)
O(1W)-Pb(1)-N(9)-N(8)	66.5(5)
C(2)-N(9)-N(8)-N(7)	−0.7(8)
Pb(1)-N(9)-N(8)-N(7)	−173.7(5)
N(9)-C(2)-N(6)-N(7)	0.2(8)
N(5)-C(2)-N(6)-N(7)	178.8(6)
N(9)-C(2)-N(6)-Pb(1)[#2]	−176.7(7)
N(5)-C(2)-N(6)-Pb(1)[#2]	1.9(13)
N(9)-N(8)-N(7)-N(6)	0.9(8)
C(2)-N(6)-N(7)-N(8)	−0.7(8)
Pb(1)[#2]-N(6)-N(7)-N(8)	177.7(5)

Symmetry transformations used to generate equivalent atoms: [#1] x+1/2,−y+3/2,z+1/2; [#2] x−1/2,−y+3/2,z−1/2.

Table S6. Hydrogen bonds for $[Pb(bta)\cdot 2H_2O]_n$ (Å and °).

D-H...A	d(D-H)	d(H...A)	d(D...A)	<(DHA)
N(5)-H(5A)...N(8) [#1]	0.880	2.164	3.032	168.63
O(1W)-H(1WB)...N(4)[#2]	0.892	1.856	2.732	166.87
O(1W)-H(1WB)...N(3)[#2]	0.892	2.563	3.411	159.00
O(1W)-H(1WA)...N(2)[#3]	0.842	2.068	2.841	152.36
O(1W)-H(1WA)...N(3)[#3]	0.842	2.666	3.228	125.41
O(2W)-H(2WB)...O(1W)[#4]	0.850	1.994	2.715	142.12
O(2W)-H(2WA)...N(3)[#5]	0.934	2.099	2.856	137.28

Symmetry transformations used to generate equivalent atoms: [#1] x−1/2,−y+3/2, z−1/2; [#2]−x,−y+2,−z+1;[#3]−x+1/2,y−1/2, −z+3/2;[#4] x−1,y,z;[#5]−x−1/2,y−1/2,−z+3/2.

Table S7. Calculated parameters used in the detonation reactions.

Parameters	Calculated Value
$C_2H_5N_9O_2Pb$ (hartree)	−20249.71
Pb (hartree)	−19527.7344
H_2O (hartree)	−76.3776
C(hartree)	37.738
N_2(hartree)	−109.447
NH_3(hartree)	−56.5045
ΔE_{det}(hartree)	0.639233
ΔE_{det}(kcal·g^{-1})	1.012072941
ΔH_{det}(kcal·g^{-1})	1.186606204
ΔH_{det}(kcal·cm^{-3})	3.856470164

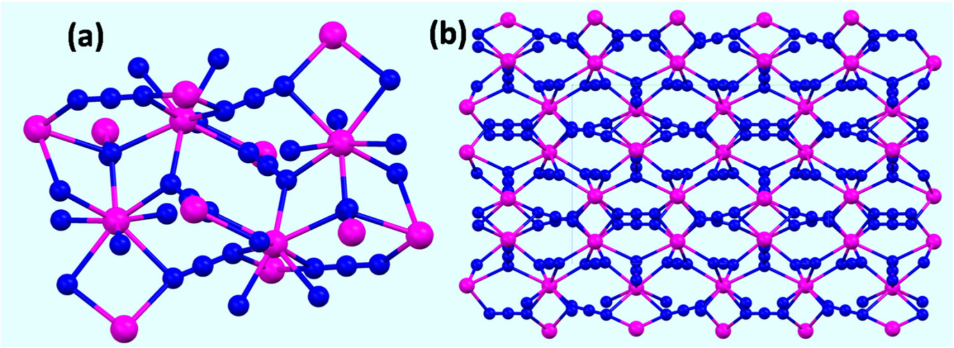

Figure S1. Ball-and-stick molecular structure (**a**) and packing diagram view down *a* axis (**b**) ofPb(N_3)$_2$.

Table S8. Crystal data and structure refinement forPb(N_3)$_2$.

Crystal Parameters	Data
Formula	$N_{18}Pb_3$
M(g/mol)	873.75
Crystal Color	colorless
Crystal System	Orthorhombic
Space Group	Pnma
a(Å)	6.6439(6)
b (Å)	16.2938(14)
c(Å)	11.3421(9)
α(°)	90
β(°)	90
γ(°)	90
V (Å^3)	1227.83(18)
Z	4
T(k)	150(2)
λ(Å)	0.71073
ρ_{calcd}(g/cm^{-3})	4.727
μ (mm^{-1})	41.065
F (000)	1488
crystal size (mm^3)	0.220×0.200×0.180
θ(°)	2.19 to 24.99
No. Refl. collected	5714
No. Indep. reflections	1117
(R_{int})	0.0575
GOF^a on F^2	1.084
$R_1{}^b$(I>2σ(I))	0.0657
w $R_2{}^c$(All refl.)	0.1869
CCDC number	1484868

^{a} GOF= Goodness of Fit;$^b R_1 = \sum||F_o| - |F_c||/\sum|F_o|$;$^c \omega R_2 = [(\omega(F_o^2 - F_c^2)^2)/\omega(F_o^2)^2]^{1/2}$.

Table S9. Atomic coordinates (×10^4) and equivalent isotropic displacement parameters (Å^2× 10^3) for $Pb(N_3)_2$.U(eq) is defined as one third of the trace of the orthogonalized U_{ij} tensor.

	x	y	z	U(eq)
Pb(1)	9392(1)	7500	8645(1)	15(1)
Pb(2)	13,386(1)	4109(1)	8769(1)	15(1)
N(1)	3370(30)	7500	9070(20)	32(6)
N(2)	4830(30)	7500	9601(17)	22(5)
N(3)	6400(30)	7500	10,136(16)	19(5)
N(4)	10,050(20)	6278(10)	10,067(13)	31(4)
N(5)	11,600(20)	5903(9)	10,001(13)	21(4)
N(6)	13,090(20)	5519(9)	9976(12)	21(3)
N(7)	11,490(20)	6331(10)	7142(13)	27(4)
N(8)	11,072(19)	5658(9)	7379(12)	14(3)
N(9)	10,656(18)	4979(9)	7636(12)	19(3)
N(10)	11,520(20)	3223(10)	7203(12)	24(4)
N(11)	11,520(30)	2500	7216(16)	13(4)

Table S10. Bond lengths (Å) and angles(°) for $Pb(N_3)_2$.

Bonds/Angles	Length(Å)/Angle(°)
Pb(1)-N(4)	2.600(15)
Pb(1)-N(3)	2.609(17)
Pb(1)-N(1)$^{\#2}$	2.69(2)
Pb(2)-N(10)	2.603(14)
Pb(2)-N(9)$^{\#3}$	2.612(13)
Pb(2)-N(9)	2.636(13)
Pb(2)-N(6)	2.682(14)
Pb(2)-N(4)$^{\#4}$	2.713(15)
Pb(2)-N(10)$^{\#3}$	2.763(14)
N(1)-N(2)	1.14(3)
N(1)-Pb(1)$^{\#5}$	2.69(2)
N(2)-N(3)	1.21(3)
N(4)-N(5)	1.20(2)
N(4)-Pb(2)$^{\#4}$	2.713(15)
N(5)-N(6)	1.173(19)
N(7)-N(8)	1.16(2)
N(8)-N(9)	1.178(19)
N(9)-Pb(2)$^{\#6}$	2.612(13)
N(10)-N(11)	1.179(16)
N(10)-Pb(2)$^{\#6}$	2.763(14)
N(11)-N(10)$^{\#7}$	1.179(16)
N(4)$^{\#1}$-Pb(1)-N(4)	100.0(8)
N(4)$^{\#1}$-Pb(1)-N(3)	74.1(4)
N(4)-Pb(1)-N(3)	74.1(4)
N(4)$^{\#1}$-Pb(1)-N(1)$^{\#2}$	73.9(5)
N(4)-Pb(1)-N(1)$^{\#2}$	73.9(5)
N(3)-Pb(1)-N(1)$^{\#2}$	129.2(6)
N(10)-Pb(2)-N(9)$^{\#3}$	99.2(5)
N(10)-Pb(2)-N(9)	68.8(5)
N(9)$^{\#3}$-Pb(2)-N(9)	79.0(3)
N(10)-Pb(2)-N(6)	142.0(4)
N(9)$^{\#3}$-Pb(2)-N(6)	83.6(4)

Table S10. *Cont.*

Bonds/Angles	Length(Å)/Angles(°)
N(9)-Pb(2)-N(6)	74.8(4)
N(10)-Pb(2)-N(4)#4	78.6(5)
N(9)#3-Pb(2)-N(4)#4	155.4(4)
N(9)-Pb(2)-N(4)#4	77.5(4)
N(6)-Pb(2)-N(4)#4	83.6(5)
N(10)-Pb(2)-N(10)#3	78.3(3)
N(9)#3-Pb(2)-N(10)#3	66.7(5)
N(9)-Pb(2)-N(10)#3	127.2(5)
N(6)-Pb(2)-N(10)#3	135.0(4)
N(4)#4-Pb(2)-N(10)#3	135.0(5)
N(2)-N(1)-Pb(1)#5	159(2)
N(1)-N(2)-N(3)	178(2)
N(2)-N(3)-Pb(1)	109.5(14)
N(5)-N(4)-Pb(1)	119.8(12)
N(5)-N(4)-Pb(2)#4	129.4(13)
Pb(1)-N(4)-Pb(2)#4	109.7(5)
N(6)-N(5)-N(4)	177.3(19)
N(5)-N(6)-Pb(2)	122.1(12)
N(7)-N(8)-N(9)	179.1(17)
N(8)-N(9)-Pb(2)#6	119.6(10)
N(8)-N(9)-Pb(2)	117.7(9)
Pb(2)#6-N(9)-Pb(2)	113.8(5)
N(11)-N(10)-Pb(2)	123.1(12)
N(11)-N(10)-Pb(2)#6	121.8(12)
Pb(2)-N(10)-Pb(2)#6	110.0(6)
N(10)#7-N(11)-N(10)	179(2)

Symmetry transformations used to generate equivalent atoms: #1 x,−y+3/2,z;#2 x+1,y,z;#3 x+1/2,y,−z+3/2;#4–x+2,−y+1,−z+2;#5 x−1,y,z;#6 x−1/2,y,−z+3/2;#7 x,−y+1/2,z.

Table S11. Anisotropic displacement parameters ($Å^2 \times 10^3$) for $Pb(N_3)_2$.The anisotropic displacement factor exponent takes the form: $-2 \pi^2 [h^2 a^{*2} U^{11} + ... + 2 h k a^* b^* U^{12}]$.

	U11	U22	U33	U23	U13	U12
Pb(1)	15(1)	13(1)	18(1)	0	1(1)	0
Pb(2)	20(1)	9(1)	17(1)	0(1)	0(1)	1(1)
N(1)	10(11)	52(18)	35(12)	0	−10(10)	0
N(2)	25(12)	17(12)	24(11)	0	12(10)	0
N(3)	9(9)	31(14)	17(9)	0	8(8)	0
N(4)	19(7)	40(11)	32(8)	18(7)	10(6)	15(7)
N(5)	23(9)	25(11)	16(8)	2(5)	−7(6)	0(7)
N(6)	25(8)	15(9)	23(8)	−5(6)	−12(6)	14(6)
N(7)	20(8)	20(11)	42(10)	3(8)	1(7)	−6(6)
N(8)	15(7)	10(8)	15(7)	−3(6)	−9(5)	3(6)
N(9)	21(8)	11(9)	26(7)	5(6)	−6(6)	−2(6)
N(10)	28(8)	18(10)	25(8)	−1(6)	−15(6)	3(6)
N(11)	14(10)	12(13)	12(9)	0	−5(7)	0

Table S12. Torsion angles (°) for $Pb(N_3)_2$.

**Torsion Angles**	**Angle (°)**
Pb(1)$^{\#5}$-N(1)-N(2)-N(3)	180.0(3)
N(1)-N(2)-N(3)-Pb(1)	0.0(3)
N(4)$^{\#1}$-Pb(1)-N(3)-N(2)	127.2(4)
N(4)-Pb(1)-N(3)-N(2)	-127.2(4)
N(1)$^{\#2}$-Pb(1)-N(3)-N(2)	180.000(7)
N(4)$^{\#1}$-Pb(1)-N(4)-N(5)	-103.5(15)
N(3)-Pb(1)-N(4)-N(5)	-173.7(16)
N(1)$^{\#2}$-Pb(1)-N(4)-N(5)	-33.6(15)
N(4)$^{\#1}$-Pb(1)-N(4)-Pb(2)$^{\#4}$	87.5(7)
N(3)-Pb(1)-N(4)-Pb(2)$^{\#4}$	17.3(5)
N(1)$^{\#2}$-Pb(1)-N(4)-Pb(2)$^{\#4}$	157.4(8)
Pb(1)-N(4)-N(5)-N(6)	172(42)
Pb(2)$^{\#4}$-N(4)-N(5)-N(6)	-21(44)
N(4)-N(5)-N(6)-Pb(2)	92(43)
N(10)-Pb(2)-N(6)-N(5)	12.1(18)
N(9)$^{\#3}$-Pb(2)-N(6)-N(5)	108.9(14)
N(9)-Pb(2)-N(6)-N(5)	28.7(14)
N(4)$^{\#4}$-Pb(2)-N(6)-N(5)	-50.0(14)
N(10)$^{\#3}$-Pb(2)-N(6)-N(5)	156.6(13)
N(7)-N(8)-N(9)-Pb(2)$^{\#6}$	153(96)
N(7)-N(8)-N(9)-Pb(2)	-61(97)
N(10)-Pb(2)-N(9)-N(8)	-140.0(14)
N(9)$^{\#3}$-Pb(2)-N(9)-N(8)	-35.5(11)
N(6)-Pb(2)-N(9)-N(8)	50.8(12)
N(4)$^{\#4}$-Pb(2)-N(9)-N(8)	137.5(13)
N(10)$^{\#3}$-Pb(2)-N(9)-N(8)	-84.7(13)
N(10)-Pb(2)-N(9)-Pb(2)$^{\#6}$	7.2(5)
N(9)$^{\#3}$-Pb(2)-N(9)-Pb(2)$^{\#6}$	111.7(7)
N(6)-Pb(2)-N(9)-Pb(2)$^{\#6}$	-161.9(6)
N(4)$^{\#4}$-Pb(2)-N(9)-Pb(2)$^{\#6}$	-75.2(6)
N(10)$^{\#3}$-Pb(2)-N(9)-Pb(2)$^{\#6}$	62.6(7)
N(9)$^{\#3}$-Pb(2)-N(10)-N(11)	124.1(16)
N(9)-Pb(2)-N(10)-N(11)	-161.6(17)
N(6)-Pb(2)-N(10)-N(11)	-144.5(14)
N(4)$^{\#4}$-Pb(2)-N(10)-N(11)	-80.8(16)
N(10)$^{\#3}$-Pb(2)-N(10)-N(11)	60.3(15)
N(9)$^{\#3}$-Pb(2)-N(10)-Pb(2)$^{\#6}$	-80.9(5)
N(9)-Pb(2)-N(10)-Pb(2)$^{\#6}$	-6.7(4)
N(6)-Pb(2)-N(10)-Pb(2)$^{\#6}$	10.5(10)
N(4)$^{\#4}$-Pb(2)-N(10)-Pb(2)$^{\#6}$	74.2(5)
N(10)$^{\#3}$-Pb(2)-N(10)-Pb(2)$^{\#6}$	-144.7(6)
Pb(2)-N(10)-N(11)-N(10)$^{\#7}$	-142(86)
Pb(2)$^{\#6}$-N(10)-N(11)-N(10)$^{\#7}$	66(88)

Symmetry transformations used to generate equivalent atoms: $^{\#1}$x,−y+3/2,z;$^{\#2}$x+1,y,z;$^{\#3}$x+1/2,y,−z+3/2;$^{\#4}$–x+2,−y+1,−z+2;$^{\#5}$ x−1,y,z;$^{\#6}$ x−1/2,y,−z+3/2;$^{\#7}$ x,−y+1/2,z.

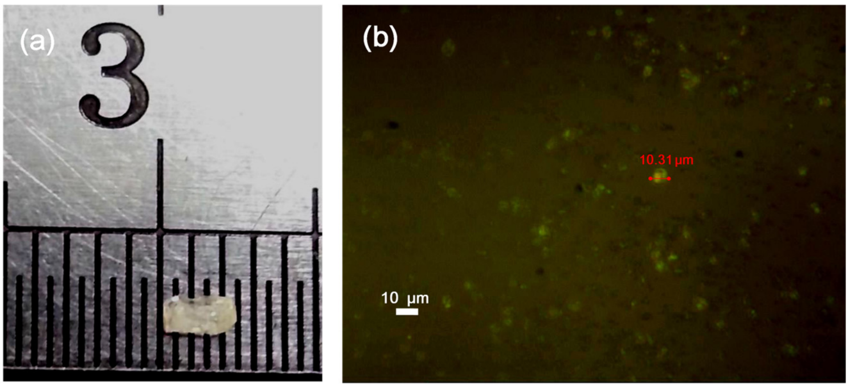

Figure S2. The pictures of single crystal MOF (a) and micron sized crystal MOF (b).

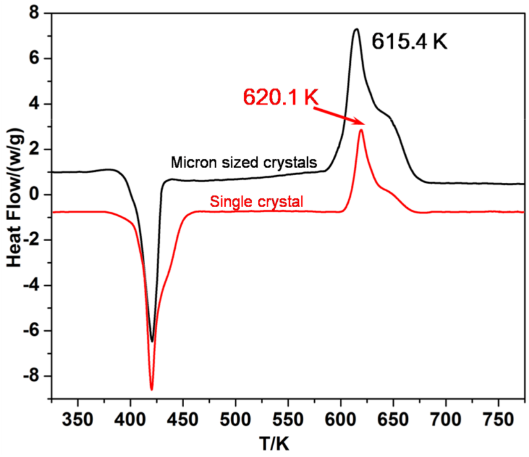

Figure S3. DSC curves of single crystal MOF (red) and micron sized crystal MOF (black) at the heating rate of 10K min^{-1}.

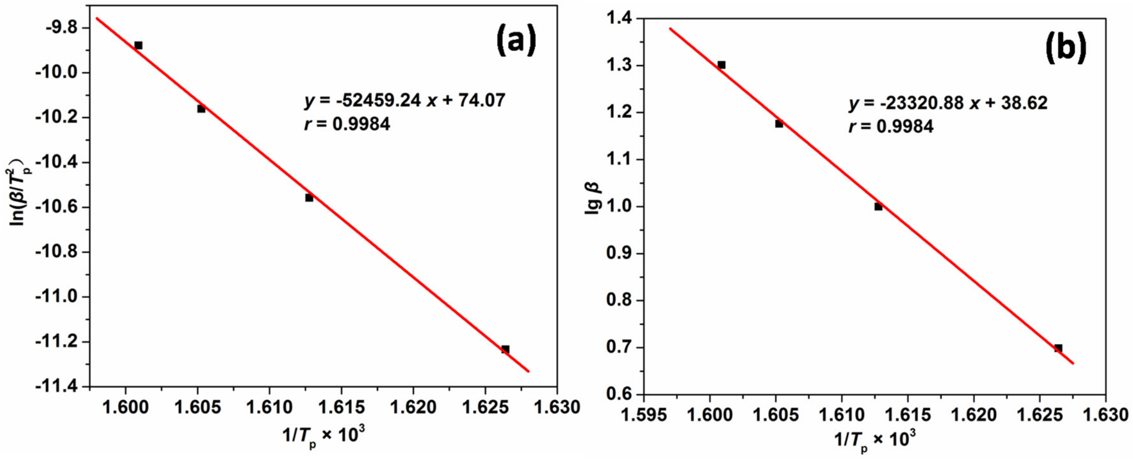

Figure S4. Linear relationship of ln (β/T_p^2) and lg (β) vs. $1/T_p$: (**a**)Kissinger's method; (**b**) Ozawa's methods.
